# Pharmacological sex hormone manipulation as a risk model for depression

**DOI:** 10.1002/jnr.24632

**Published:** 2020-05-12

**Authors:** Vibe G. Frokjaer

**Affiliations:** ^1^ Neurobiology Research Unit and Center for Integrated Molecular Brain Imaging Copenhagen Denmark; ^2^ Psychiatric Center Copenhagen Copenhagen University Hospital Rigshospitalet Copenhagen Denmark

## Abstract

Sex hormone transition may trigger severe depressive episodes in some women. In order to map mechanisms related to such phenomena we developed a pharmacological preclinical human model using sex hormone manipulation with gonadotropin releasing hormone agonist (GnRHa) in a placebo‐controlled design. Here the findings from this model is synthesized and discussed in the context of related literature on hormonal contributions to reproductive mental health disorders. The GnRha model work points to an estradiol‐dependent depressive response in healthy women undergoing short‐term sex hormone manipulation with GnRHa, which is linked to serotonin transporter changes (a key regulator of synaptic serotonin), a disengagement of hippocampus, and overengagement of brain networks recruited when processing emotional salient information. Further, the GnRHa model suggest that key brain regions in the reward circuit are less engaged in positive stimuli when undergoing sex hormone manipulation, which may underlie anhedonia. Also, the work supports that enhanced sensitivity to estrogen signaling at the level of gene expression may drive increased risk for depressive symptoms when exposed to sex steroid hormone fluctuations. In conclusion, the GnRHa model work highlights the brain signatures of rapid and profound changes in sex steroid hormone milieu, which reflect plausible mechanisms by which risk for mood disorders works. This model points to the role of estrogen dynamics and sensitivity, and offers a rationale for personalized prevention in hormonal transition phases, for example pregnancy to postpartum transition, perimenopause, and hormone treatments, which now can move into clinical translation and ideally pave the way for protecting mental and cognitive health.

## 1 INTRODUCTION

Major depressive disorder (MDD) is expected to cause the highest ranking disability and burden of disease by 2030 (WHO, 2008) and strikingly affects twice as many women as men. Women are at a particularly heightened risk during hormonal transition phases such as during puberty (Thapar, Collishaw, Pine, & Thapar, 2012), across late pregnancy to postpartum (Munk‐Olsen, Laursen, Pedersen, Mors, & Mortensen, 2006) and perimenopause (Freeman, Sammel, Boorman, & Zhang, 2014). Hormone‐related risk mechanisms may even extend to exogenous hormone exposure (i.e., hormonal contraception) (Skovlund, Morch, Kessing, & Lidegaard, 2016).

MDD is a heterogeneous and complex disorder and depressive symptoms often occur transdiagnostically, for example in other disorders such as bipolar disorder, schizophrenia, neurodegenerative, and disorders. The etiological contributions to MDD are from a multitude of environmental and genetic factors, and their interplay, which indeed can be modified by steroid hormones. In line with this, steroid hormone dynamics play a prominent role specifically in MDD compared to other psychiatric disorders, that is schizophrenia, autism spectrum‐, bipolar disorder, and alcoholism, as recently emphasized by gene expression profiles in a large postmortem brain study (Gandal et al., 2018). Yet, the underlying risk and resilience mechanisms of MDD are far from clear and accordingly current preventive and treatment strategies are suboptimal. Identification of high‐risk individuals with distinct etiology and/or responsiveness to certain triggers or treatments is a strategy to stratify and assist build a rationale for personalized and precise treatment and prevention (Schumann et al., 2014). We and others propose that one such clinically important and distinct subgroup within MDD is women who are sensitive to hormonal transitions.

In this review, we synthesize and discuss findings from a preclinical human pharmacological sex hormone manipulation model we used in healthy women to provide unique insights to how sex hormone fluctuations may trigger depressive symptoms in some but not other women (Fisher et al., 2017; Frokjaer et al., 2015; Henningsson et al., 2015; Macoveanu et al., 2016; Mehta et al., 2018; Stenbaek, Budtz‐Jorgensen, Pinborg, Jensen, & Frokjaer, 2019; Stenbaek et al., 2016).

### 1.1 Epidemiology of depressive episodes during women's hormonal transitions

Strong epidemiological evidence suggests that women are at increased risk for depressive episodes in phases of life were endogenous sex steroid hormone milieu changes, such as across late pregnancy to postpartum or menopausal transition (Lokuge, Frey, Foster, Soares, & Steiner, 2011; Munk‐Olsen et al., 2006). This includes perinatal depression (PND), which according to DSM‐V criteria is defined as a depressive episode with onset during pregnancy or up to 4 weeks postpartum. Even though the current diagnostic classifications do not distinguish between antenatal and postnatal onset of PND, there is considerable evidence pointing to critical differences, for example in genetic risk factors, which are best characterized for postpartum onset (Elwood et al., 2019). Furthermore, depressive episodes manifesting later than 4 weeks postpartum may also have hormonal contributions in their pathophysiology and the risk for depressive episodes are increased up to 5 months postpartum (Munk‐Olsen et al., 2006). PND affects 10%–20% of postpartum mothers worldwide (Fellmeth, Fazel, & Plugge, 2017; Gavin et al., 2005; Howard et al., 2014). Notably, PND may not only affect new mothers but also can, especially if untreated, be adverse for their offspring in terms of infant language and early cognitive development (Evans et al., 2012) and future health (Pearson et al., 2013; Stein et al., 2014). PND frequently has an onset in late pregnancy (Meltzer‐Brody, Boschloo, Jones, Sullivan, & Penninx, 2013) and may worsen dramatically postpartum. Intriguingly, a large seminal Danish registry study demonstrated that for new mothers, the risk for developing a mental disorder which necessitates admission to psychiatric hospital or outpatient clinic peaks early postpartum at days 10 to 19 and for unipolar depression is sustained until 5 months after birth (Munk‐Olsen et al., 2006), which coincides with the dramatic postpartum decline in placenta‐produced hormones that are built up during pregnancy. However, PND frequently has an onset in late pregnancy. Likewise, non‐pathological manifestation of transient mental distress, postpartum blues, temporally coincides with the drop in placenta‐produced steroid hormones and heightens the risk for postpartum depressive episodes (O'Hara & Wisner, 2014). On the other hand, while new fathers also may experience depressive symptoms, severe adverse mental health responses to fatherhood that necessitates admission to psychiatric hospital are *not* increased 0–12 months postpartum (Munk‐Olsen et al., 2006). This emphasizes qualitative differences between mothers and fathers in the nature of postpartum/parenthood transition and its consequences, and further support hormonal contributions to perinatal mental disorders.

Menopausal transition is another female life phase where ovarian sex steroid hormone levels fluctuate dramatically. At this time, women face about two to fourfold increased risk for depressive episodes (Bromberger et al., 2011; Freeman, Sammel, Liu, et al., 2004). Interestingly, the strongest risk factor for developing depressive symptoms across menopausal transition is fluctuation of estradiol around the women's own mean level (Freeman, Sammel, Lin, & Nelson, 2006). Notably, at the time the postmenopausal state is fully established and estradiol no longer fluctuates, that risk decreases (Freeman et al., 2014). Also, in PND, sensitivity to estradiol fluctuations seemingly is central to risk. This was demonstrated in a small (*N* = 8*2) but seminal study by Bloch et al. (2000) showing that women with a history of postpartum depression were differentially sensitive to mood‐destabilizing effects of ovarian steroids, that is estradiol and progesterone particularly in the withdrawal phase but also present in a hormone‐stimulated phase (modelling pregnancy). Recent studies have pointed to molecular mechanisms, in terms of gene expression and epigenetic modifications, of such sensitivity to ovarian hormone changes, in particular estrogen sensitivity (Guintivano, Arad, Gould, Payne, & Kaminsky, 2014; Mehta et al., 2014). Finally, the ovarian hormone sensitivity hypothesis is indirectly strengthened by observations in women with premenstrual dysphoric disorder (PMDD) who indeed develop depressive symptoms when exposed to ovarian steroid hormone replacement after a period of hormonal suppression (Schmidt et al., 2017), which again seem to involve epigenetic mechanisms related to estradiol (Marrocco et al., 2018).

With the pharmacological gonadotropin‐releasing hormone agonist (GnRHa) risk model we wanted to illuminate how hormone transitions increase the risk for triggering depressive episodes, and in particular we were interested in the role of estradiol dynamics. The GnRHa model setup we applied introduces a biphasic estradiol fluctuation (initially a stimulation and subsequently a suppression of the hypothalamus–pituitary–gonadal [HPG] axis), which enabled us to focus at the estradiol fluctuation‐driven changes and their coupling to early depressive‐like symptoms in certain susceptible women, a point of strength over previous study designs, which have more focused at the late suppression phase in recovered patients (Bloch et al., 2000).

## 2 THE GnRHa TRIAL SET‐UP

A detailed description of the trial and study population is available in Frokjaer et al. (2015). The study is registered at ClinicalTrials.gov (ID: NCT02661789). A brief overview is presented below.

### 2.1 GnRHa is a pharmacological tool to manipulate sex steroids

Pharmacologic intervention with continuous GnRHa, as can be obtained by an implant, induces a biphasic ovarian hormone response (Thomas, Jenkins, Lenton, & Cooke, 1986); after an initial stimulation of the HPG axis, pituitary GnRHa receptors desensitize, and consequently, ovarian sex steroid production is suppressed to menopausal levels within about 10 to 14 days and is sustained for 28 days. The GnRHa model thus mimics hormonal fluctuations and best matches the menopausal transition stage (Harlow et al., 2012) and reflects partly the physiological changes across the prepartum to postpartum transition where placenta‐produced hormones, including estradiol, built up in pregnancy decline rapidly from the high levels established during pregnancy.

### 2.2 Study participants

Sixty‐three healthy women (mean age 24.3 ± 4.9 years) were enrolled in this block randomized, placebo‐controlled, and double‐blinded intervention study. Two women could not complete the study program at follow‐up due to anovulation and pregnancy, respectively. Block randomization was performed to balance the distribution of 5‐HTTLPR genotype (LALA or not). All participants (mean age 24.3 ± 4.9 years) had regular menstrual cycles (duration 23–35 days). Participants were screened by face‐to‐face interview, gynecological ultrasound examinations, and blood tests to secure no significant neurological, psychiatric, endocrinological, or gynecological disorders.

### 2.3 Intervention

**FIGURE 1:**
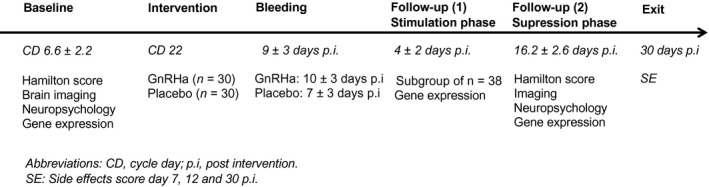
GnRHa model study design and timings

Baseline assessments were performed in the midfollicular phase (cycle day 6.6 ± 2.2) when ovarian hormone levels are most stable and time since the postovulatory estradiol drop is maximal. Contingent upon ovulation in their natural cycle, participants received a subcutaneous injection of a GnRHa implant (ZOLADEX, a biodegradable copolymer impregnated with 3.6 mg of goserelin; AstraZeneca, London, United Kingdom) (*n* = 30) or saline (*n* = 30), that is in the midluteal phase cycle day 22.6 ± 2.5, by a gynecologist not involved in any subsequent interaction with the participants, data collection, or analysis. This timing allowed a near‐identical timing of menstrual bleeding (placebo group) and withdrawal bleeding (GnRHa group), which enabled blinding. Follow‐up was placed post‐bleeding at a time point late enough to allow the GnRHa group to have entered their early ovarian suppression phase (16.2 ± 2.6 days after intervention). An overview of the timing of baseline, intervention, and follow‐up relative to the menstrual cycle is provided in Figure 1.

### 2.4 Outcomes

Data from the following domains of interest were collected at baseline and at follow‐up: (a) clinical outcomes (Hamilton depression rating scale (HDRS) of 17 items and self‐reported psychometrics), (b) serotonin transporter (SERT) brain binding ([11C]DASB PET‐scan), (c) cognitive processing of emotions (fMRI faces task), (d) reward processing (fMRI gambling task), (e) functional connectivity (resting‐state fMRI), (f) basic cognition (verbal affective memory and reaction time), and (g) gene expression and DNA methylation changes across intervention.

Also at baseline, self‐reported NEO Personality Inventory‐Revised personality trait scores were filled online to derive the neuroticism score. Neuroticism was considered relevant, since it is a robust risk factor for developing major depression that might interact with other risk factors in the interplay with markers of serotonin signaling as shown previously (Frokjaer, Vinberg, et al., 2010). Serial measurements of Profile of Mood States (POMS) were available across the intervention period as specified in Stenbaek et al. (2016).

For a subgroup of the latter 38 participants enrolled, an attempt was made to characterize the magnitude of ovarian hormone increase in the initial stimulatory phase of the GnRHa by adding hormones measurements at the day of intervention (midluteal phase) and 3 to 5 days after intervention (stimulated if GnRHa). At these time points, material for gene expression was also collected while DNA was only available from baseline and (late) follow‐up (Mehta et al., 2018).

## 3 CLINICAL OUTCOMES AND BRAIN SIGNATURES OF SEX ‐HORMONE MANIPULATIONS WITH GnRHa

The data presented below are published and discussed in detail in the corresponding original articles (Fisher et al., 2017; Frokjaer et al., 2015; Henningsson et al., 2015; Macoveanu et al., 2016; Mehta et al., 2018; Stenbaek et al., 2016, 2019). Here we provide a short synthesis and integrated discussion of the findings.

### 3.1 Summary of the GnRHa findings

As summarized in Table 1, GnRHa intervention induced depressive symptoms that approached the level of a mild depressive state in about 13% of the healthy women who participated in the study, while the remaining participants experienced more subtle or no depressive symptoms (Frokjaer et al., 2015). This number aligns well with the known frequencies of PND of around 10%–20% (Fellmeth et al., 2017; Gavin et al., 2005; Howard et al., 2014). Interestingly, only by observer‐dependent methods (semi‐structured Hamilton 17‐item interview) were the increased levels of depressive symptoms evident (Stenbaek et al., 2016). However, self‐reported serial day‐to‐day changes in total mood disturbances (by POMS) showed labile mood in the GnRHa group only in women with elevated levels of mood disturbances at baseline (Stenbaek et al., 2016). Further analyses showed that mood disturbances were most pronounced during day 0 to 5 corresponding to the early stimulation phase of the intervention and was dependent on neuroticism levels such that most extreme neuroticism scores (high or low) were most sensitive to GnRHa intervention relative to placebo (Stenbaek et al., 2019).

**TABLE 1:** Main findings from the GnRHa clinical trial

Article	*N*	Main outcomes	Main findings
Frokjaer et al. (2015)	30 GnRHa	Changes from baseline in: HDRS 17‐item Serotonin transporter BPnd Estradiol	GnRHa induced depressive symptoms (HDRS 17‐item) in about 13% relative to placebo
	30 placebo		
			No main effects of GnRHa versus placebo on serotonin transporter BPnd
			The emergence of depressive symptoms was associated with *both* increased serotonin transporter binding in neocortex from baseline and with the magnitude of estradiol decrease
Henningsson et al. (2015)	26 GnRHa	Emotional faces fMRI Amygdala (primary) medial prefrontal cortex anterior cingulate cortex ventrolateral prefrontal cortex insula	No main effects of GnRHa versus placebo
	29 placebo		Women who displayed larger GnRHa‐induced increase in depressive symptoms had a larger increase in both negative and positive emotion‐elicited activity in insula (anterior)
Macoveanu et al. (2016)	26 GnRHa	Gambling fMRI, brain activation related to reward	GnRHa reduced activation reward‐related activation of amygdala, relative to placebo
	29 placebo		
Stenbæk et al. (2016)	31 GnRHa	Affective verbal memory Reaction time Self‐reported mental distress Profile of mood states	GnRHa was associated with slower reaction time and more labile mood relative to placebo
	30 placebo		
			No effects of GnRHa were seen on affective verbal memory
Fisher et al. (2017)	29 GnRHa	rs‐fMRI: functional connectivity (rs‐FC) for amygdala hippocampus anterior cingulate dorsal raphe, median raphe posterior cingulate cortex	No main effects of GnRHa versus placebo
	29 placebo		Women who displayed larger GnRHa‐induced increase in depressive symptoms had an increased amygdala–right temporal cortex rs‐FC and decreased hippocampus–cingulate rs‐FC
Mehta (2019)		A priori PND biomarker set of 116 gene transcripts (from Mehta et al. 2014)	Of the a priori defined PND predictive set of 116 genes, 19% were differentially expressed post‐GnRHa and 49% were differentially methylated relative to placebo
			Within the GnRHa group, a large proportion of PND genes were significantly associated (gene expression; DNA methylation) with changes in depressive symptoms (28%; 66%), estradiol levels (49%; 66%), and neocortex serotonin transporter binding (8%; 45%) between baseline and later follow‐up
Stenbaek, Budtz‐Jorgensen, Pinborg, Jensen, and Frokjaer (2019)	28 GnRHa	Profile of mood states day 0 to 14 post‐intervention. Neuroticism scores at baseline (NEO‐PI‐R)	GnRHa with and without concomitant infertility‐related stress heightened total mood disturbances most pronounced at the early stimulatory phase day 0–5 post‐GnRHa in a manner dependent on neuroticism scores
	27 placebo		
	37 IVF‐GnRHa		

Molecular brain imaging data showed that depressive responses to GnRHa were coupled to increases in SERT binding in neocortex suggesting a transiently reduced synaptic serotonin level and suppressed serotonin signaling (Frokjaer et al., 2015). Functional MRI with tasks probing emotional face processing pointed to a coupling between depressive responses to GnRHa and an increased involvement of anterior insula in processing emotions irrespective of emotion valence (Henningsson et al., 2015). Furthermore, resting‐state fMRI suggested that GnRHa‐induced depressive symptoms are coupled to an overengagement of amygdala and a disengagement of hippocampus in non‐goal‐oriented cognitive processes (Fisher et al., 2017). Also, task‐based fMRI using a gambling paradigm showed that GnRHa reduces brain responses to reward (Macoveanu et al., 2015). Specifically, the amygdala, which putatively helps encode the stimulus reward value in reward processing and plays a key role in reward learning, was less engaged in processing positive of stimuli pre‐ to post‐GnRHa relative to placebo. Finally, in a very recent study we showed that an a priori defined set of gene transcripts, which were differentially expressed in third trimester in women who later developed PND with postpartum onset (Mehta et al., 2014), was also associated with depressive responses to GnRHa in a manner dependent on estradiol changes and SERT changes (Mehta et al., 2018).

## 4 DISCUSSION

### 4.1 Estradiol, serotonin brain signaling and brain function

Our GnRHa data provide direct evidence for sex hormone manipulation to trigger depressive symptoms in healthy volunteers. The depressive symptoms were subtle except in two to three participants who met the clinical criteria of a mild depressive state. The emergence of depressive symptoms was coupled to increases in SERT binding (which lowers synaptic serotonin), and was dependent on the biphasic estradiol fluctuation (Frokjaer et al., 2015). This implies transiently compromised serotonin signaling in the mechanisms by which sex steroid hormone fluctuations provoke depressive symptoms in susceptible individuals. Serotonergic brain signaling is ostensibly disturbed in individuals with MDD also with postpartum onset and traditionally constitutes a key target for pharmacological treatment (di Scalea & Wisner, 2009); however, treatment success is disappointing (Rush et al., 2006). Sex hormones target serotonergic neurons and shape the adult female brain during hormonal transition periods (Barth, Villringer, & Sacher, 2015). Thus it is likely that serotonin signaling is affected during changes in sex steroid hormone milieu. In particular, estradiol potently affects the key features of the serotonin signaling system (Bethea, Lu, Gundlah, & Streicher, 2002), that is synthesis, degradation, postsynaptic receptor distribution, including induction of the main regulator of synaptic serotonin, the SERT (Lu, Eshleman, Janowsky, & Bethea, 2003; Suda, Segi‐Nishida, Newton, & Duman, 2008; Sumner et al., 2007). Also, importantly seminal studies have pointed to a role of increased enzymatic degradation of monoamines in the brain (MAO‐A activity) in postpartum depressive symptoms (Sacher et al., 2010, 2015), which would further tend to compromise not only serotonergic signaling but also other monoamines, that is dopamine and noradrenaline. Pharmacological treatments of depressive episodes occurring in relation to peripartum traditionally target SERT, that is selective serotonin reuptake inhibitors (SSRIs; Cooper, Willy, Pont, & Ray, 2007). Nevertheless, our GnRHa results and that of others (Dowlati et al., 2017) support that the brain architecture of hormonal transition includes key targetable features beyond serotonin that ostensibly contributes to an increased risk for depressive episodes most likely linked to the estradiol *withdrawal* phase.

### 4.2 Neuroprotective properties of estradiol and estradiol sensitivity

We and others have shown that estradiol affects critical domains and key brain regions (e.g., the hippocampus) known to be dysfunctional in MDD (Barth et al., 2015, 2016; Comasco et al., 2014) which putatively is linked to neuroprotective features of estradiol. Such loss of neuroprotection may play a critical role specifically in estradiol withdrawal phases, as for example postpartum. Specifically, the GnRHa model highlights key regions in the reward circuit that are less engaged in response to positive stimuli when undergoing sex hormone manipulation as imaged in the ovarian hormone suppression phase, which may drive anhedonia in depressive episodes triggered by hormonal transitions. Also, in the same model, we have shown a disengagement of hippocampus (Fisher et al., 2017), and overengagement of brain networks recruited when processing emotional salient information (Henningsson et al., 2015).

Sex steroid hormones support neuroprotection through processes potentially driven by gene transcription and epigenetic mechanisms and are likely moderated by serotonergic brain signaling. Such steroid hormone‐driven processes may explain why pregnancy reorganizes brain structures in ways that, in healthy conditions, may prepare the brain for motherhood (Hoekzema et al., 2016). In particular, estrogen affects brain structure and function, including synaptic remodeling and neurogenesis (Yankova, Hart, & Woolley, 2001) and hippocampal plasticity (Barth et al., 2016). Estrogen replacement appears to have neuroprotective properties in animal models of early menopause (Sohrabji, 2005) and affects the primary serotonin receptor subtype 2A in brain cortex (Frokjaer, Erritzoe, et al., 2010; Kugaya et al., 2003; Moses‐Kolko et al., 2003) and the SERT (Comasco, Frokjaer, & Sundstrom‐Poromaa, 2014; Lu et al., 2003), which are key regulatory proteins in the system and markers of serotonergic wiring. Further human studies support a temporary neuroprotective effect of hormonal replacement in early menopause as reflected by increased hippocampal volumes (Lord, Buss, Lupien, & Pruessner, 2008) and improved cognitive function (i.e., verbal memory; Amin et al., 2006). Likewise, in healthy pregnancy, higher levels of estradiol are related to better verbal memory, which on the contrary is not the case for pregnant women who develop depressive symptoms (Hampson et al., 2015). Notably, a recent clinical trial supports that estradiol replacement in perimenopause protects against depressive episodes relative to placebo (Gordon et al., 2018); however no tools are yet available to personalize or time such preventive strategies during life course.

In PND, recent data suggest that estradiol sensitivity predisposes women to PND, which can be demonstrated with proxy markers for estrogen sensitivity derived from blood of pregnant women (i.e., DNA methylation Guintivano et al., 2014 and gene expression Mehta et al., 2014), thus constituting a candidate biomarker. Further strengthening the estradiol sensitivity hypothesis and the candidate biomarker, RNA and DNA material from the GnRHa study (Mehta et al., 2018) points to a link between these gene transcript PND markers (Mehta et al., 2014) and estradiol manipulation, which intriguingly predicts depressive responses. Importantly, this backtranslation from clinical PND biomarkers in pregnant cohorts to the GnRHa sex hormone manipulation preclinical human model further substantiates the estradiol sensitivity hypothesis of depressive episodes triggered by hormonal transitions across reproductive female life. Finally, the fact that we now can demonstrate an overlap between changes in gene expression and DNA methylation and psychometrics between the GnRHa elicited patterns and the independent clinical cohort of women with moderate to severe depressive episodes postpartum further validates the psychopathological importance of the phenomena triggered by the GnRHa manipulation. It also support GnRHa as a means of modelling mechanisms by which hormonal transition can trigger depressive episodes in certain sensitive women.

## 5 CLINICAL PERSPECTIVES AND FUTURE DIRECTIONS

Additional studies are needed to translate findings from the GnRHa model to clinically relevant groups of women. It is *not* yet known if disturbed serotonin signaling (Frokjaer et al., 2015), brain network recruitment (Henningsson et al., 2015; Macoveanu et al., 2016; Stenbaek et al., 2016), and functional connectivity (Fisher et al., 2017) translate to women who are sensitive to hormonal transitions, for example across the transition from natural pregnancy to early postpartum in women with a history of PND. This will be a critically needed step toward informing a stratified setup for prevention and clinical management of perinatal depression and to validate potential biomarkers, that is of estradiol sensitivity that may help identify such subgroups of women.

To counterbalance risk contributions from postpartum withdrawal from estradiol, transdermal estradiol has been suggested as a promising treatment option for PND (Moses‐Kolko, Berga, Kalro, Sit, & Wisner, 2009) supported by a convergence of epidemiological, preclinical, and clinical research, that is robust and rapid response to estradiol in some pilot PPD trials, few side effects, and minimal breast milk passage to the infant (Pinheiro, Bogen, Hoxha, & Wisner, 2016). However, a pilot clinical trial attempting to evaluate the effectiveness was disappointing (Wisner et al., 2015). Intriguingly, estradiol was administered at a late time point (up to 13 weeks postpartum) where a depressive episode had already been established (Wisner et al., 2015). It is not known if short‐term estradiol applied in the immediate postpartum, as a preventive strategy in a selected high‐risk group of women, may disrupt early risk mechanisms and protect maternal brain health. Other promising new PND treatments include allopregnanolone analogues, which have shown clinical efficacy; however, notably, at the same time, a large placebo effect was observed (Meltzer‐Brody et al., 2018). Importantly, robust evidence support heterogeneity of depressive episodes across the perinatal period (Putnam et al., 2017), which needs to be embraced by researchers and clinicians to fully exploit windows of opportunity for personalized prevention and treatment for the disorder(s) (Galea & Frokjaer, 2019). Notably, the GnRHa model suggests that both an *increase* in estradiol, which is pronounced in late pregnancy, and a subsequent *withdrawal* as seen postpartum contribute critically to offset brain biology and trigger depressive symptoms in susceptible women (Fisher et al., 2017; Frokjaer et al., 2015; Henningsson et al., 2015; Macoveanu et al., 2016; Stenbaek et al., 2016). Importantly, this may explain why the risk for depressive symptoms peaks in the early postpartum phase, that is, day 10–19 (Munk‐Olsen et al., 2006) where carryover effects from late pregnancy and effects of postpartum hormonal withdrawal add up. Again this may be worsened by the effects of MAO‐A activity further depleting monoamines, including serotonin in the early postpartum (Sacher et al., 2010, 2015). Intriguingly, this also raises the hypothesis that perinatal depression with onset in late pregnancy indeed have critical contributions from disturbed serotonin signaling that, however, may not be fully compensated by SSRI treatment when entering the postpartum withdrawal phase.

Risk mechanisms for developing depression identified by the GnRHa model may identify key features of a clinical relevant “hormone‐sensitive subgroup” of the broader category of MDD. Recent evidence from studies across menopausal transition supports the notion that hormonal transitions may cause depressive symptoms in hormone‐sensitive individuals: Estradiol fluctuations around menopausal transition are associated with first‐time onset of MDD (Freeman et al., 2006) and appears to be preventable by hormonal replacement (Gordon et al., 2018). Another remarkable and recent register‐based finding, which again links exposure to hormonal transitions with depression, has shown that starting on hormonal contraceptive is associated with an increased risk of developing a depressive episode (Skovlund et al., 2016; Zettermark, Perez Vicente, & Merlo, 2018). This finding has been replicated in an independent prospective cohort study (de Wit et al., 2019). It remains unclear why the use of oral contraceptives increases the risk of a depressive episode in some women, including to what extent suppression of endogenous estradiol may play a role, and if these women can be identified by risk markers.

The GnRHa model does not align as well with sex hormone dynamics putatively underlying premenstrual dysphoric mood disorder (PMDD) since the disorder seem to be linked to sensitivity to high premenstrual levels of the active metabolite of progesterone, allopreganolone (Lanza di Scalea & Pearlstein, 2019), that most likely does not vary substantially with short‐term GnRHa manipulation. However, allopregnanolone also may be linked to serotonin brain signaling (Sundstrom Poromaa et al., 2018). Accordingly, patients with PMDD appear to respond particularly well to cyclic SSRI treatment administered prior to menses and similar to inhibition of allopregnanolone (Bixo et al., 2017). Indeed, progesterone and estradiol dynamics may interact in their effects on mental health; for example as shown in women with borderline personality disorder high levels of progesterone may render a woman more sensitive to estradiol deviations (lower than the women usual mean) and elicit emotional and mood instability, impulsivity, irritability, and aggressive behaviors (Eisenlohr‐Moul, DeWall, Girdler, & Segerstrom, 2015), which also characterize PMDD. Those sex steroid hormone interactions are not captured with the GnRHa model, which also highlights why these complex phenomena also need to be studied in clinical cohorts.

Future studies evaluating preventive strategies in women at high risk for depressive episodes triggered by hormonal transitions and candidate biomarkers such as estrogen sensitivity, which may help stratify risk, are warranted. Pharmacologically, such strategies may target serotonergic signaling in late pregnancy and/or estradiol replacement in the immediate postpartum. Other strategies may include dietary supplies that can lower MAO‐A activity (Dowlati et al., 2017). In perimenopause, preventive strategies could include a precision medicine approach to identifying women who may benefit from hormonal replacement in combination with antidepressant treatments. Also in a long‐term perspective it needs to be determined if estrogen sensitivity markers based on gene transcription profiles or epigenetics (Guintivano et al., 2014; Mehta et al., 2014, 2018) can work outside of the highly stimulated state of late pregnancy, for example via estrogen or steroid hormone‐stimulated assays and whether such markers also identify women at risk for depressive episodes when exposed to other hormonal transitions; however, this is so far unexplored.

Clearly, joint efforts to facilitate replications across data sets and sites will be needed to validate potential risk stratification and biomarker tools (Freeman, Sammel, Rinaudo, & Sheng, 2004; Guintivano, Manuck, & Meltzer‐Brody, 2018) and to optimize risk and disease management to support mental health including affective cognitive functions, that is, across perimenopause and peripartum. Ideally, advancing the understanding of hormonal contributions to depressive episodes may also help fight stigma and be useful in psychoeducation to support patient engagement in preventive initiatives and treatment compliance; however, this remains to be tested in clinical trials.

## 6 CONCLUSION

The GnRHa model, given its placebo‐controlled design and through modeling the contributions from a biphasic estradiol fluctuation, allowed us to isolate the estradiol fluctuation‐driven changes and their coupling to early depressive‐like symptoms in certain susceptible women, a point of strength over previous study designs, which have focused at the late suppression phase.

Taken together the GnRHa model provides an important source of insights into the ways by which sex hormone fluctuations can trigger depressive episodes of great translational value. The model supports that both an *increase* in estradiol and a subsequent *withdrawal* contribute critically to brain architecture of risk for depression in susceptible women who display estradiol sensitivity at a molecular level. However, the relative contributions from estradiol increase phases and subsequent dramatic decreases are not clarified and, accordingly, not yet exploited in current risk or disease management.

Our data point to a distinct pathophysiology of depressive episodes related to hormonal transitions. If better understood, and if bridging with observations in clinical cohorts, this may provide a starting point for actual preventive and personalized treatment strategies of relevant intensity to be tested in future studies.

## CONFLICT OF INTEREST

VGF declares that she has received honorarium as consultant for SAGE therapeutics.

## Supporting information

Transparent Peer Review Report
