# Supplementary material for: Pharmacological sex hormone manipulation as a risk model for depression
Source: J Neurosci Res. 2020 May 12;98(7):1283–92. doi: 10.1002/jnr.24632 (PMC7383584; doi:10.1002/jnr.24632)
Supplement: Supplementary file 1 — Transparent Peer Review Report [file JNR-98-1283-s001.docx]

**Pharmacological sex hormone manipulation as a risk model for depression**

Frokjaer,Vibe

_____________________________________________________________________________________

| Review timeline: | Submission date: 13 May 2019  Editorial Decision: 24 June 2019  Revision Received: 6 April 2020  Editorial Decision:  Revision Received:  Accepted: 11 April 2020 |  |
| --- | --- | --- |
|  |  |  |

Editor: Ghiani, Cristina

Reviewer: Osborne, Lauren

Reviewer: Schweizer, Sophie

Reviewer: Sacher, Julia

_____________________________________________________________________________________

1st Editorial Decision	24 May 2019

_____________________________________________________________________________________

Reviewer: 1

Comments to the Author
This paper is relatively well written and provides a clear synthetic discussion of an important clinical trial that has already resulted in a number of interesting papers.  My big reservation has to do with what type of paper this is.  All of the results discussed herein are previously published, so there are no new data.  But, unlike typical review papers, this one discusses only the author’s own work – so while it is not an original data paper it is also not a review paper.  Looking at the author guidelines for the journal, I see that “Authors of reviews should avoid concentrating solely or preferentially on their own work,” and also that “Commentaries and Letters to the Editor provide focused viewpoints from field leaders on specific topics of interest. These can call attention to papers of particular note or provide critical comments on previous work.”  I think this paper is more akin to a commentary or letter to the editor.  I’d therefore recommend recasting it as such – which perhaps means eliding some of the sections and shortening it slightly.  (I would probably shorten and collapse together the two sections that describe the trial, the “GnRHa trial set-up” and “Clinical outcomes and brain signatures” sections; this would be easy to do by referring readers to published articles for more details on methods and perhaps by slightly expanding the amount of detail in Table 1 and having fewer details in the text.)  Or, the author could choose to write a broader review paper that discusses her work in the context of others – that would mean substantial expansion; it could still highlight this study but would also need to draw on work done by other groups.

I have a few specific comments tied to page and line numbers:

3, ll 18-19 – worth mentioning anxiety disorders here too.  PPD, PMDD, and perimenopausal depression have significant anxiety symptoms, so not clear to me that steroid hormone fluctuations are linked more strongly to MDD than to anxiety

4 – your definition of PND is consistent with the DSM definition, but there is considerable scientific evidence pointing to PPD as biologically distinct from antenatal depression (particularly genetic risk factors).  I think you need to acknowledge this, and point out that the definition of PND you are using here is not universally accepted by researchers in the field.

5 ll 38ff – It would be helpful here to point out that the GnRHa intervention is mimicking hormone fluctuation – in other words, that what seems most relevant is the fact that hormones are changing, rather than that they are being shut down.  So while levels are suppressed within 10-14 days, the key period (and the one on which your results are based) is not that suppression, but the 10 days during which change is occurring.

6 ll 55-56 – I’m confused about the extra time point in 38 participants.  This isn’t reflected in the timeline, nor in your description of what is happening above.  Can you explain more fully and also incorporate into the timeline?  In what way are you categorizing the initial stimulatory phase?

7 ll 18-19 – The description of results from the 2015 study here doesn’t match that in Table 1.  Here, you say that GnRHa induced symptoms in about 13%, but in the table you say no difference between GnRHa and placebo.  (First line of description in table perhaps has a missing word, I can’t really understand it.) These are not mutually contradictory statements, but they imply the exact opposite, and very confusing for the reader – pick one way to describe findings and stick to it.  My reading of this paper is that there WERE differences between GnRHa and placebo.

Table 1 – Can’t figure out why some of these squares are shaded with grey.


Reviewer: 2

Comments to the Author
This is a timely and interesting contribution as it provides a coherent summary of the main findings of the GNRH model for E2 sensitivity to depressed mood and I am supportive of publication pending only minor revision.

(1) I suggest to include a main overview-figure visualising all the mechanisms implicated in this model (e.g. GnRH induced ovarian hormone fluctuations, 5-HTT changes, hippocampal and neural circuit findings, epigenetic effects), how they relate to each other to predispose some women to heightened risk for depression and ultimately trigger depressed mood.

(2) when discussing insufficient serotonergic compensation in the postpartum phase for PPD, the author may wants to include the role of increased MAO-A in PPD, which represents one mechanism affecting all monoamines and not just serotonin.

(3) it would be interesting to include more explicit thoughts on what the author recommends as the next step for the clinical translation of the GnrH model. A clinical trial with a transdermal E2 patch in PPD in the early pp period? what would be the recommended dose for such a trial, what would be the time-frame suggested? Based on the effect size found in the GnrH proof of concept, what sample size would be needed for such a trial to be informative?

(4) are there any treatment perspectives (maybe more longterm) which could be derived from the epigenetic findings of this model?

Reviewer: 3

Comments to the Author
Dear Vibe,

thank you very much for your manuscript- what a delight to be able to review this! Once again, I am reading a manuscript from you that I think is so important and exciting for the research community. Your GnRHa-model represents such a helpful design in that- among others- it allows us to look at a biphasic estradiol fluctuation. I think your MS is almost ready for publication- just a few ideas that came to my mind that may offer opportunities for making it even better. My ideas concern mainly the Clinical perspectives and Conclusion parts. I felt that the links that you have established between your model and the different reproductive mood disorders (RMDs) in these final sections to be very exciting. I wondered whether this could be expanded a bit by making clear sections on each of the RMDs that you mention and compare the links that you have established between them and your model.
In particular the arguments you make concerning the role of the increase and withdrawal of estradiol in the etiology of depressive symptoms would lend themselves here as a matter of comparison along the lines of which you could accomplish this. Maybe it could be helpful to think about/ discuss that it is also the ratios of E2/P4 that are crucial in the genesis of psychiatric disorders (as our colleague Tory Eisenlohr-Moul shows in her publication of 2015). In particular the argument that rises in E2 may only constitute a mood risk when accompanied by a decline in P4 which links the whole thing to the ALLO hypothesis of depression. I would find it extremely helpful if you could elaborate on these links in your two final sections and to set your model into the bigger picture with this. What do you think?
One more minor comment: on p. 11 line 10 I would prefer the terms emotional/ affective versus cognitive, both psychological subfields instead of mental and cognitive brain health.
I hope you find my comments helpful. Thank you again for your wonderful work!

____________________________________________________________________________________

Authors’ Response	06 April 2020

____________________________________________________________________________________

Thanks for your patience and chance to edit and resubmit this paper.

I found the majority of reviewer comments very constructive and helpful in improving the manuscript and have changed the present version as follows:

I addressed all R1 points regarding table (could not keep track changes). Addressed all other R1 specific points including revise figure 1 (pls see track changes).

I also addressed most points by R2, including expansions on MAO-A studies and perspectives also longer term. However, I did not detail a study design for evaluating one single specific preventive strategy for perinatal depression as suggested, again not to narrow the manuscript too much. Also, I did not produce an extra overview figure. The table covers an overview of the GnRHa model related potential risk mechanisms for depressive symptoms triggered by estradiol fluctuations.

Major revisions include putting the GnRH agonist model into broader context and discuss more detailed related literature, and also, as suggested by R3, expand the links to other reproductive mental health disorders in women.

The manuscript is now just under 4000 words and references are 74.

Sincerely

Vibe Frøkjær

_____________________________________________________________________________________
